# Schrödinger’s Ballot: Quantum Information and the Violation of Arrow’s Impossibility Theorem

**DOI:** 10.3390/e23081083

**Published:** 2021-08-20

**Authors:** Xin Sun, Feifei He, Mirek Sopek, Meiyun Guo

**Affiliations:** 1 Department of Foundation of Computer Science, Catholic University of Lublin, 20-950 Lublin, Poland; 2 Institute of Logic and Cognition, Sun Yat-sen University, Guangzhou 510275, China; heff5@mail2.sysu.edu.cn; 3 MakoLab SA, 91-062 Lodz, Poland; sopek@makolab.com; 4 Institute of Logic and Intelligence, Southwest University, Choingqing 400715, China; guomy007@swu.edu.cn

**Keywords:** vote, quantum information, Arrow’s Impossibility Theorem, social choice

## Abstract

We study Arrow’s Impossibility Theorem in the quantum setting. Our work is based on the work of Bao and Halpern, in which it is proved that the quantum analogue of Arrow’s Impossibility Theorem is not valid. However, we feel unsatisfied about the proof presented in Bao and Halpern’s work. Moreover, the definition of Quantum Independence of Irrelevant Alternatives (QIIA) in Bao and Halpern’s work seems not appropriate to us. We give a better definition of QIIA, which properly captures the idea of the independence of irrelevant alternatives, and a detailed proof of the violation of Arrow’s Impossibility Theorem in the quantum setting with the modified definition.

## 1. Introduction

Many voting protocols based on classical cryptography have been developed and successfully applied in the last two decades [1,2]. However, the security of protocols based on classical cryptography is based on the unproven complexity of some computational algorithms, such as the factoring of large numbers. The research in quantum computation shows that quantum computers are able to factor large numbers in a short time, which means that classical protocols based on such algorithms are already insecure. To react to the risk posed by forthcoming quantum computers, a number of quantum voting protocols have been developed in the last decade [3,4,5,6,7,8,9,10,11,12,13].

While all these works have focused on the security problems of voting from a cryptographic perspective, Bao and Halpern [14] studied quantum voting from a social choice theoretic perspective by showing that the quantum analog of Arrow’s Impossibility Theorem is violated in the quantum setting. The idea and formalization of Bao and Halpern [14] are both interesting. However, we feel unsatisfied about the proof presented in [14]. From a mathematical perspective, the proof in [14] is not rigorous, especially in dealing with the notion of Quantum independence of irrelevant alternatives (QIIA). Moreover, the definition of QIIA in [14] seems not appropriate to us since it does not capture the idea of independence of irrelevant alternatives. Facing an inappropriate definition of QIIA and an unsatisfying proof in [14], we can still question whether Arrow’s Impossibility Theorem is indeed violated in the quantum setting. To answer this question, in this paper we give a better definition of QIIA, which properly captures the idea of independence of irrelevant alternatives, and a detailed proof of the violation of Arrow’s Impossibility Theorem in the quantum setting with the modified definition.

The structure of this paper is as follows. We review some background knowledge on classical and quantum voting in Section 2. In Section 3 we introduce a voting rule called quantum Condorcet voting and prove that Arrow’s Impossibility Theorem is violated by quantum Condorcet voting. We discuss related work in Section 4 and conclude this paper with the plan for the future work in Section 5. Some primitives of the quantum information theory which are used in this paper are collected in Appendix A.

## 2. Background

### 2.1. Classical Voting System

Now we will briefly review the theory of classical voting. A more detailed introduction to the classical voting and social choice theory can be found in Zwicker [15], Pacuit [16] and Brandt et al. [17].

Let $V=\{v_{1},\ldots ,v_{n}\}$ be a finite set of (at least two) voters and $C=\{c_{1},c_{2},\ldots ,c_{m}\}$ be a non-empty set of candidates. Each voter $v_{i}\in V$ is endowed with preference ${≻}_{i}$ over C. The preference ${≻}_{i}$ is a binary relation on C that is irreflexive, transitive, and complete. In other words, ${≻}_{i}$ is a linear order on C. Set theoretically, an order ≻ is defined by a set of ordered pairs such as $\left\{\left(c_{1},c_{2}\right),\left(c_{2},c_{3}\right),\left(c_{1},c_{3}\right)\right\}$. We use x≻y to represent (x,y)∈≻. Let L(C) denote the set of all linear orders on C. A profile $R=\left(R_{1},\ldots ,R_{n}\right)\in L{\left(C\right)}^{V}$ is a vector of linear orders (i.e., preferences), where $R_{i}$ is the linear order supplied by voter $v_{i}$. We write $V_{x≻y}^{R}$ to denote the set of voters that rank candidate *x* above candidate *y* under profile R. A Social Welfare Function (SWF) is a function $F:L{\left(C\right)}^{V}\mapsto L\left(C\right)$. Two widely accepted properties of SWF are unanimity and independence of irrelevant alternatives.

**Definition** **1** (Unanimity)**.** *An SWF F satisfies the unanimity condition if, whenever all voters rank x above y, then so does society:*

$$V_{x≻y}^{R}=V\;implies\;(x,y)\in F(R).$$

**Definition** **2** (Independence of Irrelevant Alternatives (IIA))**.** *An SWF F satisfies IIA if the relative social ranking of two candidates only depends on their relative voter rankings:*

$$V_{x≻y}^{R}=V_{x≻y}^{R^{\prime }}\;implies\;\left(x,y\right)\in F\left(R\right)\Leftrightarrow \left(x,y\right)\in F\left(R^{\prime }\right).$$

The intuition about IIA is that two ballot profiles that are *similar* according to (x,y) should produce the same ranking for (x,y). This intuition will be used later to define the quantum analogue of IIA.

The celebrated Arrow’s Impossibility Theorem states that any SWF that satisfies both unanimity and IIA must also satisfy a property that any SWF should not satisfy: dictatorship.

**Definition** **3** (Dictatorship)**.** *An SWF F satisfies dictatorship if there is a voter* $v_{i}\in V$ *such that* $F\left(R\right)=R_{i}$ *for every profile* $R=(R_{1},\ldots ,R_{n})$.

**Theorem** **1** (Arrow [18])**.** *Any SWF for three or more candidates that satisfies unanimity and the IIA must also satisfy dictatorship.*

Arrow’s Impossibility Theorem is an important result in the field of social choice and welfare economics. According to the theorem, when there are more than two options, it is impossible for a ranked-voting system to reach a community-wide order of preferences by collecting and converting individuals’ preferences orders while meeting a set of conditions which are the requirements for a reasonably fair voting procedure.

### 2.2. Quantum Voting System

Now we introduce our formalism of quantum voting system, which is similar to the formalism of Bao and Halpern [14]. In a quantum voting system with candidates $C=\{c_{1},c_{2},\ldots ,c_{n}\}$, we specify a Hilbert space H of which the dimension is |L(C)|. That is, $H=C^{\left|L(C)\right|}$. Every voter $v_{i}\in V$ is associated with a Hilbert space $H_{i}$ which is isomorphic to H. Every linear order R∈L(C) is naturally viewed as basis vector |R〉 of H. The basis $B={\{|R^{1}\rangle }_{i},\ldots ,|R^{\left|L(C)\right|}\rangle_{i}\}$ is called the preference basis for $H_{i}$.

Consider a pair (x,y) of candidates. H decomposes into subspaces associated with the possible relationships between *x* and *y*. By $S^{x≻y}$, we denote the subspace spanned by the B elements that encode x≻y (e.g., |x≻y≻z〉, |z≻x≻y〉). We use $\Pi^{x≻y}$ to denote the projector onto the subspace $S^{x≻y}$ and use $\Omega^{x≻y}$ to denote the maximal mixed state of the subspace $S^{x≻y}$, i.e., $\Omega^{x≻y}=\frac{1}{\mathrm{Tr}(\Pi^{x≻y})}\Pi^{x≻y}$.

A quantum ballot of voter $v_{i}$ is a density operator $\rho \in D(H_{i})$. A quantum ballot profile is a density operator $\rho \in D(H_{1}⊗\ldots ⊗H_{n})$. We use ${\mathrm{Tr}}_{\neq i}\left(\rho \right)$ to denote the quantum ballot obtained by applying partial trace on ρ to trace out all components that are not equal to *i*. A basis quantum ballot profile is a profile in which every component is a density operator of a basis vector. A quantum social welfare function (QSWF) is a linear map $E:D\left(H_{1}⊗\ldots ⊗H_{n}\right)\mapsto D\left(H\right)$. The result of voting with quantum ballot profile ρ is obtained by measuring E(ρ) on the preference basis.

**Definition** **4** (Quantum Unanimity [14])**.** *A QSWF E satisfies the sharp unanimity condition if it satisfies the following:*

- *For all quantum ballot profiles ρ and all pairs of candidates (x,y), if $\mathrm{Tr}(\Pi^{x≻y}\left({\mathrm{Tr}}_{\neq i}\left(\rho \right)\right))=1$ for each voter $v_{i}$, then $\mathrm{Tr}(\Pi^{x≻y}\left(E\left(\rho \right)\right))=1$.*

*A QSWF E satisfies the unsharp unanimity condition if it satisfies the following:*

- *For all quantum ballot profiles ρ and all pairs of candidates (x,y), if $\mathrm{Tr}(\Pi^{x≻y}\left({\mathrm{Tr}}_{\neq i}\left(\rho \right)\right))>0$ for each voter $v_{i}$, then $\mathrm{Tr}(\Pi^{x≻y}\left(E\left(\rho \right)\right))>0$.*

*A QSWF E satisfies the quantum unanimity condition if it satisfies both sharp and unsharp unanimity conditions.*

Sharp unanimity ensures that if all voters prefer *x* to *y* with certainty, then the society prefers *x* to *y* with certainty. On the other hand, unsharp unanimity ensures that if every voter prefers *x* to *y* with positive probability, then the society also prefers *x* to *y* with positive probability.

**Definition** **5** (Quantum Independence of Irrelevant Alternatives (QIIA))**.** *A QSWF E satisfies the sharp IIA condition if it satisfies the following:*

- *For all quantum ballot profiles ρ and $\rho^{\prime }$ and all pairs of candidates (x,y), if $\mathrm{Tr}\left(\Pi^{x≻y}\left({\mathrm{Tr}}_{\neq i}\left(\rho \right)\right)\right)=\mathrm{Tr}\left(\Pi^{x≻y}\left({\mathrm{Tr}}_{\neq i}\left(\rho^{\prime }\right)\right)\right)$ for each voter $v_{i}$, then $\mathrm{Tr}(\Pi^{x≻y}\left(E\left(\rho \right)\right))=1$ implies that $\mathrm{Tr}(\Pi^{x≻y}\left(E\left(\rho^{\prime }\right)\right))=1$.*

*A QSWF E satisfies unsharp IIA if the following condition is satisfied:*

- *For all quantum ballot profiles $\rho ,\rho^{\prime }$ and all pairs of candidates (x,y), if $\mathrm{Tr}\left(\Pi^{x≻y}\left({\mathrm{Tr}}_{\neq i}\left(\rho \right)\right)\right)=\mathrm{Tr}\left(\Pi^{x≻y}\left({\mathrm{Tr}}_{\neq i}\left(\rho^{\prime }\right)\right)\right)$ for each voter $v_{i}$, then $\mathrm{Tr}(\Pi^{x≻y}\left(E\left(\rho \right)\right))>0$ implies that $\mathrm{Tr}(\Pi^{x≻y}\left(E\left(\rho^{\prime }\right)\right))>0$.*

*A QSWF E satisfies the QIIA condition if it satisfies both sharp and unsharp IIA conditions.*

Note that QIIA in our definition is different from the QIIA in [14]. QIIA in [14] states that whether E(ρ) has support on $S^{x≻y}$ depends only on whether each $\rho_{i}$ has support on $S^{x≻y}$ and $S^{y≻x}$. More precisely, it states that for all quantum ballot profiles $\rho ,\rho^{\prime }$ and all pairs of candidates (x,y), if $\mathrm{Tr}(\Pi^{x≻y}\left({\mathrm{Tr}}_{\neq i}\left(\rho \right)\right))>0$ iff $\mathrm{Tr}(\Pi^{x≻y}\left({\mathrm{Tr}}_{\neq i}\left(\rho^{\prime }\right)\right))>0$ and $\mathrm{Tr}(\Pi^{y≻x}\left({\mathrm{Tr}}_{\neq i}\left(\rho \right)\right))>0$ iff $\mathrm{Tr}(\Pi^{y≻x}\left({\mathrm{Tr}}_{\neq i}\left(\rho^{\prime }\right)\right))>0$ for each voter $v_{i}$, then $\mathrm{Tr}(\Pi^{x≻y}\left(E\left(\rho \right)\right))>0$ implies that $\mathrm{Tr}(\Pi^{x≻y}\left(E\left(\rho^{\prime }\right)\right))>0$.

The intuition of QIIA is the same as the intuition of classical IIA: it states that two ballot profiles that are similar according to (x,y) should produce the same ranking for (x,y). It seems Bao and Halpern [14] considered two ballot profiles ρ and $\rho^{\prime }$ to be similar according to (x,y) as long as $\mathrm{Tr}(\Pi^{x≻y}\left({\mathrm{Tr}}_{\neq i}\left(\rho \right)\right))>0$ iff $\mathrm{Tr}(\Pi^{x≻y}\left({\mathrm{Tr}}_{\neq i}\left(\rho^{\prime }\right)\right))>0$ and $\mathrm{Tr}(\Pi^{y≻x}\left({\mathrm{Tr}}_{\neq i}\left(\rho \right)\right))>0$ iff $\mathrm{Tr}(\Pi^{y≻x}\left({\mathrm{Tr}}_{\neq i}\left(\rho^{\prime }\right)\right))>0$. To us this requirement is too weak. For example, ρ=0.99|x≻y≻z〉〈x≻y≻z|+0.01|y≻x≻z〉〈y≻x≻z| and $\rho^{\prime }=0.01|x≻y≻z\rangle \langle x≻y≻z|+0.99|y≻x≻z\rangle \langle y≻x≻z|$ are similar according to (x,y) in Bao and Halpern’s definition, but intuitively they shouldn’t. On the other hand, in our definition ρ and $\rho^{\prime }$ are not similar according to (x,y). Indeed, in our definition two profiles ρ and $\rho^{\prime }$ are similar according to (x,y) only if $\mathrm{Tr}\left(\Pi^{x≻y}\left({\mathrm{Tr}}_{\neq i}\left(\rho \right)\right)\right)=\mathrm{Tr}\left(\Pi^{x≻y}\left({\mathrm{Tr}}_{\neq i}\left(\rho^{\prime }\right)\right)\right)$ for all voter $v_{i}$. According to our QIIA, ρ and $\rho^{\prime \prime }=0.99|x≻z≻y\rangle \langle x≻z≻y|+0.01|y≻z≻x\rangle \langle y≻z≻x|$ are similar according to (x,y). We believe our definition of QIIA properly captures the idea of independence of irrelevant alternatives. That’s why we use it to replace the QIIA of Bao and Halpern [14].

**Definition** **6** (Quantum Dictatorship [14]). *A QSWF E satisfies sharp dictatorship if there is a voter $v_{i}$ such that:*

- *For all quantum ballot profiles $\rho =(\rho_{1},\ldots ,\rho_{n})$ and all pairs of candidates (x,y), $\mathrm{Tr}(\Pi^{x≻y}\rho_{i})=1$ iff $\mathrm{Tr}(\Pi^{x≻y}\left(E\left(\rho \right)\right))=1$.*

*A QSWF E satisfies unsharp dictatorship if there is a voter $v_{i}$ such that:*

- *For all quantum ballot profiles $\rho =(\rho_{1},\ldots ,\rho_{n})$ and all pairs of candidates (x,y), $\mathrm{Tr}(\Pi^{x≻y}\rho_{i})>0$ iff $\mathrm{Tr}(\Pi^{x≻y}\left(E\left(\rho \right)\right))>0$.*

*A QSWF E satisfies quantum dictatorship if it satisfies both sharp and unsharp dictatorship.*

Sharp dictatorship states that whenever the dictator prefers *x* to *y* with certainty, then so does the society. Unsharp dictatorship states that whenever the dictator prefers *x* to *y* with positive probability, then so does the society.

## 3. Quantum Condorcet Voting and Arrow’s Impossibility Theorem

We will use a special voting rule called Quantum Condorcet Voting $E_{qcv}$ to refute Arrow’s Impossibility Theorem in the quantum setting. Since $E_{qcv}$ is a linear map from $D(H_{1}⊗\ldots ⊗H_{n})$ to D(H), we only need to specify how $E_{qcv}$ operates on a basis quantum ballot profile.

**Definition** **7** (Quantum Condorcet Voting)**.** *Let $\rho_{1}⊗\ldots ⊗\rho_{n}$ be a basis quantum ballot profile. The quantum Condorcet voting $E_{qcv}$ operates in the following steps:*

*1.*  *Calculates the Condorcet score of each candidate according to $\rho_{1}⊗\ldots ⊗\rho_{n}$. The Condorcet score of a candidate is the number of winning in pairwise comparison with other candidates. That is, for a candidate x, his Condorcet score $S_{c}\left(x\right)$ is $\left|\{y\in C:|V_{x≻y}^{R}|\geq |V_{y≻x}^{R}|\}\right|$ where R is the classical ballot profile corresponding to $\rho_{1}⊗\ldots ⊗\rho_{n}$.*
*2.*  *Generate a weak order ⪰ over all candidates according to their Condorcet score. That is, x⪰y iff $S_{c}\left(x\right)\geq S_{c}\left(y\right)$.*
*3.*  *Complete the weak order. That is, generate the set $\left\{{≻}^{1},\ldots ,{≻}^{m}\right\}$ in which each ${≻}^{i}$ is a linear order that extends ⪰ and $\left\{{≻}^{1},\ldots ,{≻}^{m}\right\}$ contains all extensions of ⪰.*
*4.*  *Transform the linear order into a quantum state. That is, for $\left\{{≻}^{1},\ldots ,{≻}^{m}\right\}$ we create a quantum state $\sigma^{1}=\frac{1}{m}\sum_{i}\sigma_{i}$, where each $\sigma_{i}$ is the basis ballot that corresponds to ${≻}^{i}$.*
*5.*  *Give the minority a shot. For any candidate pair (x,y) which is encoded by at least one $\rho_{i}$, We spread an amount δ∈(0,1) of weight across the x≻y subspace. That is, $\sigma^{1}$ is changed to $\sigma^{2}=\left(1-k\delta \right)\sigma^{1}+\delta \Omega^{x_{1}≻y_{1}}+\ldots +\delta \Omega^{x_{k}≻y_{k}}$, where $\left(x_{1},y_{1}\right),\ldots ,\left(x_{k},y_{k}\right)$ ranges over all candidate pairs that are encoded by at least one $\rho_{i}$. The parameter δ is required to satisfy that $\delta <\frac{1}{{\left|C\right|}^{2}}$.*
*6.*  *Enforce unanimity. For any candidate pair (x,y) which is encoded by all the $\rho_{i}$, we project $\sigma^{2}$ onto the x≻y subspace. That is, $\sigma^{2}$ is changed to $\sigma^{3}=\frac{\Pi^{x_{k}≻y_{k}}\ldots \Pi^{x_{1}≻y_{1}}\sigma^{2}\Pi^{x_{1}≻y_{1}}\ldots \Pi^{x_{k}≻y_{k}}}{\mathrm{Tr}(\Pi^{x_{k}≻y_{k}}\ldots \Pi^{x_{1}≻y_{1}}\sigma^{2})}$, where $\left(x_{1},y_{1}\right),\ldots ,\left(x_{k},y_{k}\right)$ ranges over all candidate pairs that are encoded by all the $\rho_{i}$.*

Both *giving the minority a shot* and *enforcing unanimity* are first introduced in Bao and Halpern [14]. While they may look strange at first sight, both of them will be useful in disproving Arrow’s Impossibility Theorem.

**Theorem** **2.** *The Quantum Condorcet Voting $E_{qcv}$ satisfies sharp unanimity.*

**Proof:**  Let $\rho =\rho_{1}⊗\ldots ⊗\rho_{n}$ be a basis quantum ballot profile. If $\mathrm{Tr}(\Pi^{x≻y}\left(\rho_{i}\right))=1$ for each voter $v_{i}$, then each $\rho_{i}$ encodes x≻y since $\rho_{i}$ is a basis ballot. Then the projector $\Pi^{x≻y}$ will be applied in the step of enforcing unanimity. Therefore, $\mathrm{Tr}(\Pi^{x≻y}\left(E_{qcv}\left(\rho \right)\right))=1$.

Now let ρ be a quantum ballot profile such that $\mathrm{Tr}(\Pi^{x≻y}\left({\mathrm{Tr}}_{\neq i}\left(\rho \right)\right))=1$ for each voter $v_{i}$. Note that $\mathrm{Tr}(\Pi^{x≻y}\left({\mathrm{Tr}}_{\neq i}\left(\rho \right)\right))=1$ implies that ${\mathrm{Tr}}_{\neq i}\left(\rho \right)=x_{1}^{i}|\phi_{1}^{i}\rangle \langle \phi_{1}^{i}|+\ldots +x_{m}^{i}|\phi_{m}^{i}\rangle \langle \phi_{m}^{i}|$ where each $\phi_{j}^{i}$ is a basis vector that encodes x≻y and $\sum_{j}x_{j}^{i}=1$. Therefore, $\rho =x_{1}^{1}\ldots x_{1}^{n}|\phi_{1}^{1}⊗\ldots ⊗\phi_{1}^{n}\rangle \langle \phi_{1}^{1}⊗\ldots ⊗\phi_{1}^{n}|+\ldots +x_{m}^{1}\ldots x_{m}^{n}|\phi_{m}^{1}⊗\ldots ⊗\phi_{m}^{n}\rangle \langle \phi_{m}^{1}⊗\ldots ⊗\phi_{m}^{n}|$. It then follows that $\mathrm{Tr}(\Pi^{x≻y}({\mathrm{Tr}}_{\neq i}(|\phi_{j}^{1}⊗\ldots ⊗\phi_{j}^{n}\rangle \langle |\phi_{j}^{1}⊗\ldots ⊗\phi_{j}^{n}\left|))\right)=1$ for all *i*. Then we know that $\mathrm{Tr}(\Pi^{x≻y}(E_{qcv}(|\phi_{j}^{1}⊗\ldots ⊗\phi_{j}^{n}\rangle \langle |\phi_{j}^{1}⊗\ldots ⊗\phi_{j}^{n}\left|))\right)=1$ because $|\phi_{j}^{1}⊗\ldots ⊗\phi_{j}^{n}\rangle \langle |\phi_{j}^{1}⊗\ldots ⊗\phi_{j}^{n}|$ is a basis quantum ballot profile. Therefore, we have $\mathrm{Tr}\left(\Pi^{x≻y}\left(E_{qcv}\left(\rho \right)\right)\right)=x_{1}^{1}\ldots x_{1}^{n}+\ldots +x_{m}^{1}\ldots x_{m}^{n}=1$. □

**Theorem** **3.** *The Quantum Condorcet Voting $E_{qcv}$ satisfies unsharp unanimity.*

**Proof:**  Let $\rho =\rho_{1}⊗\ldots ⊗\rho_{n}$ be a basis quantum ballot profile where each $\rho_{i}$ is a basis vector of $H_{i}$. If $\mathrm{Tr}(\Pi^{x≻y}\left(\rho_{i}\right))>0$ for each voter $v_{i}$, then each $\rho_{i}$ encodes x≻y since $\rho_{i}$ is a basis ballot. Then the projector $\Pi^{x≻y}$ will be applied in the step of enforcing unanimity. Therefore, $\mathrm{Tr}(\Pi^{x≻y}\left(E_{qpv}\left(\rho \right)\right))=1>0$.

Now let ρ be a quantum ballot profile such that $\mathrm{Tr}(\Pi^{x≻y}\left({\mathrm{Tr}}_{\neq i}\left(\rho \right)\right))>0$ for each voter $v_{i}$. Note that $\mathrm{Tr}(\Pi^{x≻y}\left({\mathrm{Tr}}_{\neq i}\left(\rho \right)\right))>0$ implies that ${\mathrm{Tr}}_{\neq i}\left(\rho \right)=x_{i}|\phi_{i}\rangle \langle \phi_{i}|+\ldots$ for some basis vector $|\phi_{i}\rangle$ which encodes x≻y and $0<x_{i}\leq 1$. Hence $\rho =x_{1}\ldots x_{n}|\phi_{1}⊗\ldots ⊗\phi_{n}\rangle \langle \phi_{1}⊗\ldots ⊗\phi_{n}|+\ldots$. Note that $|\phi_{1}⊗\ldots ⊗\phi_{n}\rangle \langle \phi_{1}⊗\ldots ⊗\phi_{n}|$ is a basis quantum ballot profile in which each $\phi_{i}$ encode x≻y. It then follows that $\mathrm{Tr}(\Pi^{x≻y}\left(E_{qcv}(|\phi_{1}⊗\ldots ⊗\phi_{n}\rangle \langle \phi_{1}⊗\ldots ⊗\phi_{n}|)\right))=1$. From $0<x_{1}\ldots x_{n}\leq 1$ we now know that $\mathrm{Tr}(\Pi^{x≻y}\left(E_{qpv}\left(\rho \right)\right))>0$. □

**Theorem** **4.** *The Quantum Condorcet Voting $E_{qcv}$ satisfy sharp IIA.*

**Proof:**  Let $\rho =\rho_{1}⊗\ldots ⊗\rho_{n}$ and $\rho^{\prime }=\rho_{1}^{\prime }⊗\ldots ⊗\rho_{n}^{\prime }$ be two basis quantum ballot profiles. Assume $\mathrm{Tr}\left(\Pi^{x≻y}\left(\rho_{i}\right)\right)=\mathrm{Tr}\left(\Pi^{x≻y}\left(\rho_{i}^{\prime }\right)\right)$ for each voter $v_{i}$. If $\mathrm{Tr}(\Pi^{x≻y}\left(E_{qcv}\left(\rho \right)\right))=1$, then we know x≻y is encoded by all the $\rho_{i}$. For otherwise $\Omega^{y≻x}$ will appear in $\sigma^{2}$ in the step of giving the minority a shot, making $\mathrm{Tr}(\Pi^{x≻y}\left(E_{qcv}\left(\rho \right)\right))<1$. Since $\mathrm{Tr}\left(\Pi^{x≻y}\left(\rho_{i}\right)\right)=\mathrm{Tr}\left(\Pi^{x≻y}\left(\rho_{i}^{\prime }\right)\right)$ for each voter $v_{i}$, we know that x≻y is encoded by all the $\rho_{i}^{\prime }$. Hence $\mathrm{Tr}(\Pi^{x≻y}\left(E_{qcv}\left(\rho^{\prime }\right)\right))=1$.

Now, let ρ and $\rho^{\prime }$ be quantum ballot profiles such that $\mathrm{Tr}\left(\Pi^{x≻y}\left({\mathrm{Tr}}_{\neq i}\left(\rho \right)\right)\right)=\mathrm{Tr}\left(\Pi^{x≻y}\left({\mathrm{Tr}}_{\neq i}\left(\rho^{\prime }\right)\right)\right)$ for each voter $v_{i}$. If $\mathrm{Tr}(\Pi^{x≻y}\left(E_{qcv}\left(\rho \right)\right))=1$, then we know x≻y is encoded by all ${\mathrm{Tr}}_{\neq i}\left(\rho \right)$, i.e., $\mathrm{Tr}(\Pi^{x≻y}({\mathrm{Tr}}_{\neq i}($ρ)))=1. For otherwise $\Omega^{y≻x}$ will appear in $\sigma^{2}$ in the step of giving the minority a shot and $\Pi^{x≻y}$ will not appear in $\sigma^{3}$ in the step of enforcing unanimity, making $\mathrm{Tr}(\Pi^{x≻y}\left(E_{qcv}\left(\rho \right)\right))<1$. Since $\mathrm{Tr}(\Pi^{x≻y}\left({\mathrm{Tr}}_{\neq i}\left(\rho \right)\right))$$=\mathrm{Tr}(\Pi^{x≻y}\left({\mathrm{Tr}}_{\neq i}\left(\rho^{\prime }\right)\right))$ for each voter $v_{i}$, we know that x≻y is encoded by all $\rho_{i}^{\prime }$. Hence $\mathrm{Tr}(\Pi^{x≻y}(E_{qcv}(\rho^{\prime }$)))=1. □

**Theorem** **5.** *The Quantum Condorcet Voting $E_{qcv}$ satisfies unsharp IIA.*

**Proof**:  Let $\rho =\rho_{1}⊗\ldots ⊗\rho_{n}$ and $\rho^{\prime }=\rho_{1}^{\prime }⊗\ldots ⊗\rho_{n}^{\prime }$ be two basis quantum ballot profiles. Assume $\mathrm{Tr}\left(\Pi^{x≻y}\left(\rho_{i}\right)\right)=\mathrm{Tr}\left(\Pi^{x≻y}\left(\rho_{i}^{\prime }\right)\right)$ for each voter $v_{i}$. From $\mathrm{Tr}(\Pi^{x≻y}\left(E_{qcv}\left(\rho \right)\right))>0$ we know that y≻x is not encoded by all candidates. Without loss of generality, let’s assume $\rho_{1}$ encodes x≻y but not y≻x. Then $\rho_{1}^{\prime }$ also encodes x≻y but not y≻x. Hence $\mathrm{Tr}(\Pi^{x≻y}\left(E_{qcv}\left(\rho^{\prime }\right)\right))>0$.

Now, let ρ and $\rho^{\prime }$ be quantum ballot profiles such that $\mathrm{Tr}\left(\Pi^{x≻y}\left({\mathrm{Tr}}_{\neq i}\left(\rho \right)\right)\right)=\mathrm{Tr}\left(\Pi^{x≻y}\left({\mathrm{Tr}}_{\neq i}\left(\rho^{\prime }\right)\right)\right)$. If $\mathrm{Tr}(\Pi^{x≻y}\left(E_{qcv}\left(\rho \right)\right))>0$, then we know y≻x is not encoded by all ${\mathrm{Tr}}_{\neq i}\left(\rho \right)$. Since $\mathrm{Tr}\left(\Pi^{x≻y}\left({\mathrm{Tr}}_{\neq i}\left(\rho \right)\right)\right)=\mathrm{Tr}\left(\Pi^{x≻y}\left({\mathrm{Tr}}_{\neq i}\left(\rho^{\prime }\right)\right)\right)$ for each voter $v_{i}$, we know that y≻x is not encoded by all ${\mathrm{Tr}}_{\neq i}\left(\rho^{\prime }\right)$. Hence $\mathrm{Tr}(\Pi^{x≻y}($ $E_{qcv}\left(\rho^{\prime }\right)))>0$. □

**Theorem** **6.** *The Quantum Condorcet Voting $E_{qcv}$ does not satisfy sharp dictatorship.*

**Proof:**  We will construct a ballot profile in which no candidate is a dictator. Let {x,y,z} be the set of candidates. Let $\rho =\rho_{1}⊗\rho_{2}⊗\rho_{3}$ be a quantum ballot profile where $\rho_{1}=|x≻y≻z\rangle \langle x≻y≻z|,\rho_{2}=|y≻z≻x\rangle \langle y≻z≻x|,\rho_{3}=|z≻x≻y\rangle \langle z≻x≻y|$. Then the weak order generated by $E_{qcv}$ according to the Condorcet score is x≡y≡z. The completion of x≡y≡z is {x≻y≻z,x≻z≻y,y≻x≻z,y≻z≻x,z≻x≻y,z≻y≻x}. Therefore the quantum state generated in step 4 of quantum Condorcet voting is $\sigma^{1}=\frac{1}{6}(|x≻y≻z\rangle \langle x≻y≻z|+|x≻z≻y\rangle \langle x≻z≻y|+|y≻x≻z\rangle \langle y≻x≻z|+|y≻z≻x\rangle \langle y≻z≻x|+|z≻x≻y\rangle \langle z≻x≻y|+|z≻y≻x\rangle \langle z≻y≻x|)$. Therefore, we have $\mathrm{Tr}(\Pi^{x≻y}\rho_{1})=1$ but $\mathrm{Tr}(\Pi^{x≻y}\left(E_{qcv}\left(\rho \right)\right))<1$, $\mathrm{Tr}(\Pi^{y≻z}\rho_{2})=1$ but $\mathrm{Tr}(\Pi^{y≻z}\left(E_{qcv}\left(\rho \right)\right))<1$, $\mathrm{Tr}(\Pi^{z≻x}\rho_{3})=1$ but $\mathrm{Tr}(\Pi^{z≻x}\left(E_{qcv}\left(\rho \right)\right))<1$. This violates sharp dictatorship. □

**Theorem** **7.** *The Quantum Condorcet Voting $E_{qcv}$ does not satisfy unsharp dictatorship.*

**Proof:**  Consider again the profile constructed in the above proof. We have $\mathrm{Tr}(\Pi^{z≻x}\left(E_{qcv}\left(\rho \right)\right))>0$ but $\mathrm{Tr}(\Pi^{z≻x}\rho_{1})≯0$, $\mathrm{Tr}(\Pi^{x≻y}\left(E_{qcv}\left(\rho \right)\right))>0$ but $\mathrm{Tr}(\Pi^{x≻y}\rho_{2})≯0$, $\mathrm{Tr}(\Pi^{y≻z}\left(E_{qcv}\left(\rho \right)\right))>0$ but $\mathrm{Tr}(\Pi^{y≻z}\rho_{3})≯0$. This violates unsharp dictatorship. □

By combining Theorems 2–7 we conclude that Quantum Condorcet Voting satisfies Quantum Unanimity and the QIIA but prevents Quantum Dictatorship. In other words, we can infer the following corollary:

**Corollary** **1.** *Arrow’s Impossibility Theorem is not valid in quantum voting.*

## 4. Related Work

### 4.1. Security of Quantum Voting

Most of the related work on quantum voting focus on the security of voting. The first quantum voting protocol was proposed by Hillery et al. [3]. They proposed two voting modes, namely traveling ballot and distributed ballot to ensure the security of voting. The protocol designed by Vaccaro et al. [4] uses entangled states to ensure that the votes are anonymous and to allow the votes to be tallied. The entanglement is distributed over separated sites; the physical inaccessibility of any one site is sufficient to guarantee the anonymity of the votes. Horoshko and Kilin [6] proposed a quantum anonymous voting scheme based on a Bell-state. Their protocol protects both the voters from a curious tallyman and all the participants from a dishonest voter in an unconditionally secure way. Wang et al. [10] proposed a quantum anonymous voting protocol assisted by two kinds of entangled quantum states. They provided a mechanism of opening and permuting the ordered votes of all the voters in an anonymous manner; any party who is interested in the voting results can obtain the voting result through a simple calculation. Their protocol possesses the properties of privacy, self-tallying, nonreusability, verifiability, and fairness at the same time.

In our previous work [13] a simple voting protocol based on Quantum Blockchain was proposed. Despite its simplicity, our protocol satisfies the most important properties of the secure voting protocols: is anonymous, binding, non-reusable, verifiable, eligible, fair and self-tallying. The protocol could also be implemented using presently available technology. One limitation of this protocol is that it works for only 2 candidates. In a recent paper [19] we overcame that limitation by realizing the classical Condorcet voting on Quantum Blockchain.

### 4.2. Probabilistic Social Choice

Another field of research related to ours is the probabilistic social choice theory [20,21]. In probabilistic social choice, a voter’s ballot is represented by a probability distribution $\left(p_{1},\ldots ,p_{m}\right)$ over candidates $C=\{c_{1},\ldots ,c_{m}\}$, where $p_{i}$ is the probability for the voter to vote for $c_{i}$. The voting rules in probabilistic social choice is a function that maps a collection of ballots to a social ballot, which is again a probability distribution over candidates C.

Classical ballot and probabilistic ballot are incomparable in the sense that one cannot completely express the other. It is easy to see that classical ballot cannot express probabilistic ballot. On the other hand, although it is shown by Intriligator [20] that a probabilistic ballot induces a weak order on candidates simply by ranking them according to the probability assigned to them, this order is by no means a classical ballot. Indeed, a classical ballot x≻y≻z informs us that *x* is chosen with certainty when comparing *x* and *y* and comparing *x* and *z*, *y* is chosen with certainty when comparing *y* and *z*. But after some simple deduction we can convince ourselves that no probabilistic ballot can give us the same information.

Quantum ballot unifies both classical and probabilistic ballots as special cases. A basis quantum ballot |x≻y〉 is the same as a classical ballot x≻y. For a probabilistic ballot $\left(p_{x},p_{y},p_{z}\right)$, the quantum ballot $p_{x}|x≻y≻z\rangle \langle x≻y≻z|+p_{y}|y≻x≻z\rangle \langle y≻x≻z|+p_{z}\left|z≻x≻y\rangle \langle z≻x≻y\right|$ is one of its quantum analogues.

The classical Arrow’s theorem, often implicitly, assumes that the social welfare function should yield a unique and complete ranking of societal choices for any set of individual voter preferences. Therefore, it must provide the same ranking each time voters’ preferences are presented the same way (i.e., deterministically). This is usually referred to as universality. I am not sure if this condition is fulfilled in the quantum approach.

## 5. Conclusions and Future Work

In this paper we study Arrow’s Impossibility Theorem in the quantum setting. We first modify the definition of QIIA in a way that precisely captures the idea of independence of irrelevant alternatives. We then present a detailed proof of the violation of Arrow’s Impossibility Theorem with our modified definition.

The violation of Arrow’s Impossibility Theorem shows that quantum voting outperforms classical voting in practice from the perspective of democracy. The existing work on quantum voting has already demonstrated its advantage on security. Since quantum voting has advantages in both democracy and security, we believe that quantum voting machines may be deployed for election in many countries in the foreseeable future with the advancement of quantum information technology.

In [19], we have demonstrated that Condorcet voting on Quantum Blockchain significantly simplifies the task of electronic voting, and at the same time ensures many desired security properties. In the future, we will further improve Quantum Condorcet Voting such that it has advantages for both security and the quality of the democratic processes.

We will also investigate the validity of other theorems of classical social choice theory in the quantum setting. Those theorems include Sen’s Theorem on the impossibility of a Paretian Liberal [22], the Muller-Satterthwaite Theorem on surjective monotonicity [23] and the Gibbard-Satterthwaite Theorem on strategic manipulation [24]. The third direction of research we are interested in, is quantum logic for social choice. Modal logic has been used as a powerful tool to model and reason about social choice [25,26,27,28]. It is both natural and valuable to develop a quantum logic to model and reason about quantum social choice. A category theoretic characterization of Arrow’s Impossibility Theorem was given in Abramsky [29]. Concerning the usage of category theory in the research of quantum information [30], we will also develop a category theoretic characterization of quantum voting in the future.

The classical social welfare function is deterministic in the sense that it yields a unique ranking of societal choices for any set of ballot profiles. Therefore, it provides the same ranking each time voters’ preferences are presented the same way. This is usually referred to as universality. Does universality hold in quantum voting? It seems the answer is both yes and no. Universality holds in quantum voting in the sense that a quantum social welfare function yields a unique quantum ballot for a given quantum ballot profile. Universality does not hold in quantum voting in the sense that measuring a quantum ballot in the preference basis produces a basis ballot, which is the final result of voting, in a non-deterministic manner. This observation suggests that universality is not a proper concept in quantum voting. In the future we will study some variants of universality which plays a meaningful role in quantum voting.

## Data Availability

No datasets were generated or analysed during the current study.

## Appendix A. Basics of Quantum Information

Some primitives of quantum information which are used in this paper are collected in this appendix. The readers who are interested in quantum information are recommended to textbooks such as Yanofsky and Mannucci [31], Scherer [32], Nielsen and Chuang [33] and Watrous [34].

**Definition** **A1** (Hilbert space)**.** *A (finite-dimensional) Hilbert space H is a*

1. *complex vector space, that is,*
  ϕ,ψ∈H and a,b∈C ⇒aϕ+bψ∈H,
2. *with a (positive-definite) scalar product 〈·|·〉:H×H↦C such that for all $\phi ,\psi ,\phi_{1},\phi_{2}\in H$ and a,b∈C*
  (a) $\langle \phi |\psi \rangle =\bar{\langle \psi |\phi \rangle }$
  (b) 〈ϕ|ϕ〉≥0
  (c) *〈ϕ|ϕ〉=0 iff ϕ=0*
  (d) $\langle \psi |a\phi_{1}+b\phi_{2}\rangle =a\langle \psi |\phi_{1}\rangle +b\langle \psi |\phi_{2}\rangle$

In quantum computation and quantum information, we only consider finite-dimensional Hilbert spaces. A vector ϕ of a Hilbert space is usually represented in the Dirac notion as |ϕ〉 in quantum computing. A Hilbert space H induces a norm ∥.∥ defined by $∥\phi ∥=\sqrt{\langle \phi |\phi \rangle }$ for any ϕ∈H.

**Definition** **A2** (orthonormal basis and dimension)**.** *An orthonormal basis $\left\{|\phi_{i}\rangle \right\}$ for a Hilbert space H is a basis of H whose vectors are unit vectors and are orthogonal to each other, that is, for any $|\phi_{i}\rangle ,|\phi_{j}\rangle$, $∥\phi_{i}∥=1$ and $\langle \phi_{i}|\phi_{j}\rangle =0$. The dimension of a Hilbert space is the number of vectors of an orthonormal basis.*

**Definition** **A3** (tensor product)**.** *Given Hilbert spaces V and W of dimension m and n respectively, their tensor product, denoted V⊗W, is a mn-dimensional space consisting of linear combinations of outer products |v〉⊗|w〉 of vectors $|v\rangle ={\left(v_{1},v_{2},\cdots ,v_{m}\right)}^{T}\in V$ and $|w\rangle ={\left(w_{1},w_{2},\cdots ,w_{n}\right)}^{T}\in W$, where*

(A1) $$|v\rangle ⊗|w\rangle =\left[\begin{array}{c} v_{1}w_{1}\\ v_{1}w_{2}\\ \vdots \\ v_{m}w_{n} \end{array}\right]$$

**Definition** **A4** (subspace)**.** *A subspace of a Hilbert space V is a subset W of V such that W is also a Hilbert space.*

**Definition** **A5** (operator)**.** *A linear map A:H↦H is called an operator on H.*

We use L(H) to denote the set of all operators on H.

**Definition** **A6** (adjoint)**.** *The operator $A^{*}:H\mapsto H$ that satisfies $\langle A^{*}\phi |\psi \rangle =\langle \phi |A\psi \rangle$ for all ϕ,ψ∈H is called the adjoint operator to A.*

**Definition** **A7** (projector)**.** *A projector of a Hilbert space H is a linear map P:H↦H such that $P^{2}=P$ and $P^{*}=P$.*

Every subspace *V* corresponds to a unique projector $P_{V}$. Projectors are related to projective measurements in quantum mechanics. We use an operator *M* to represent an observable of the quantum system being observed, with a decomposition $M=\sum_{m}P_{m}$, where $P_{m}$ is the projector onto the eigenspace of *M* with eigenvalue *m*. The result of measuring the state |ψ〉 will be one of *M*’s eigenvalues, and the probability of getting result *m* is $p\left(m\right)=\langle \psi |P_{m}|\psi \rangle$.

**Definition** **A8** (trace)**.** *Let H be a Hilbert space and ρ be an operator on H. The trace of ρ is defined by*

$$\mathrm{Tr}\left(\rho \right)=\sum_{i}\langle i|\rho |i\rangle$$

*where {|i〉} is an orthonormal basis of H.*

**Definition** **A9** (positive semidefinite operator)**.** *An operator A:H↦H is positive semidefinite if it holds that $A=B^{*}B$ for some operator B∈L(H).*

**Definition** **A10** (density operator)**.** *A positive semidefinite operator ρ on H is a density operator if it holds that $\rho =\rho^{*}$ and Tr(ρ)=1.*

**Definition** **A11** (partial trace)**.** *Suppose the composite system of two subsystems A and B is described by the density operator $\rho_{AB}$. The partial trace over B is defined by*

$$\rho_{A}={\mathrm{Tr}}_{B}\left(\rho_{AB}\right)=\sum_{i}\left(I_{A}⊗\langle i|)\rho_{AB}(I_{A}⊗|i\rangle \right)$$

*where {|i〉} is an orthonormal basis of the Hilbert space $H_{B}$. $\rho_{A}$ is called the reduced density operator of the subsystem A. The partial trace over A can be defined in a similar way.*
